# Tenofovir induced renal damage is associated with activation of NF-κB inflammatory signaling pathway and PARP overactivation

**DOI:** 10.1186/1471-2334-14-S3-E5

**Published:** 2014-05-27

**Authors:** Hemalatha Ramamoorthy, Bina Isaac, Premila Abraham

**Affiliations:** 1 Department of Biochemistry, Christian Medical College, Bagayam, Vellore, Tamilnadu, India; 2 Department of Anatomy, Christian Medical College, Bagayam, Vellore, Tamilnadu, India

## Background

Tenofovir is recommended as a first-line therapy in HIV treatment. However, its long term use is associated with proximal tubular injury and renal dysfunction. Tenofovir has been shown to target the proximal tubular mitochondria, resulting in severe mitochondrial injury and overproduction of ROS and RNS. ROS are potent stimuli for the activation of NF-κB, a key transcription factor, which is known to mediate inflammation.The NF-κB response proinflammatory genes include iNOS, COX, TNFα, and others such as PARP-1. In the present study, we investigated whether NF-κB inflammatory signaling pathway plays a role in tenofovir nephrotoxicity.

## Methods

Rats were administered 2 daily doses of tenofovir (300mg/kg body weight) by gavage for 35 consecutive days, while control rats received water alone. On the 36^th^ day, the rats were killed and the kidneys were used for the following assays. (a)NFkB protein expression by WB and immunostaining methods, mRNA expression by RT-PCR and activity by ELISA, and IkB-α protein expression and mRNA expression, (b) iNOS protein expression by WB, immunostaining methods, and mRNA expression by PCR, (c) COX-2 protein and mRNA expression, and (d) PARP-1 protein.

## Results

The results of the present study reveal that NF-κB signaling pathway is upregulated in the kidneys of rats chronically treated with tenofovir, as evidenced by a statistically significant increase in the mRNA and protein expressions of NFkB, and its target proinflammatory genes, iNOS, COX-2, TNF α and PARP-1 as compared with control.

## Conclusion

NF-κB inflammatory signaling pathway may play a role in the pathophysiology of tenofovir nephrotoxicity.

